# TSS Removal Efficiency and Permeability Degradation of Sand Filters in Permeable Pavement

**DOI:** 10.3390/ma16113999

**Published:** 2023-05-26

**Authors:** Phuong T.-H. Nguyen, Jongyeong Kim, Jaehun Ahn

**Affiliations:** Department of Civil and Environmental Engineering, Pusan National University, Busan 46241, Republic of Korea

**Keywords:** permeable pavement, TSS, TSS particle size, TSS concentration, hydraulic gradient, permeability degradation, TSS removal efficiency

## Abstract

Permeable pavement is a highly effective technology in Low-Impact Development (LID) for managing stormwater runoff, which helps mitigate environmental impacts. Filters are essential components of permeable pavement systems as they prevent permeability reduction, remove pollutants, and enhance the system’s overall efficiency. This research paper focuses on exploring the influence of three factors, including total suspended solids (TSS) particle size, TSS concentration, and hydraulic gradient, on the permeability degradation and TSS removal efficiency of sand filters. A series of tests were conducted using different values of these factors. The results demonstrate that these factors have an influence on permeability degradation and TSS removal efficiency (TRE). A larger TSS particle size results in higher permeability degradation and TRE than a smaller particle size. Higher TSS concentrations lead to higher permeability degradation and lower TRE. Additionally, smaller hydraulic gradients are associated with higher permeability degradation and TRE. However, the influence of TSS concentration and hydraulic gradient seems less significant than that of TSS particle size for the values of the factors considered in the tests. In summary, this study provides valuable insights into the effectiveness of sand filters in permeable pavement and identifies the main factors that influence permeability degradation and TRE.

## 1. Introduction

Increased infrastructure and development have led to a reduction in natural surface cover, altering the water cycle and causing severe flooding in urban areas [1]. In addition, runoff from urban areas contributes to the majority of pollutants transported to various water bodies [2]. Therefore, it is necessary to provide strategies to mitigate the disadvantages of imperviousness in modern cities [3]. One of the technological developments associated with Low-Impact Development (LID) is the permeable pavement system, which has been shown to be an effective tool in addressing runoff-related problems [4]. Permeable pavement can be an excellent alternative to impervious surfaces such as roads, parking lots, and walkways [5,6]. Permeable paving materials are specifically designed to facilitate the infiltration and storage of rainwater [7,8]. There are several types of permeable pavement, such as permeable interlocking concrete pavement (PICP), porous asphalt pavement (PAP), and pervious concrete pavement (PCP).

**Figure 1:**
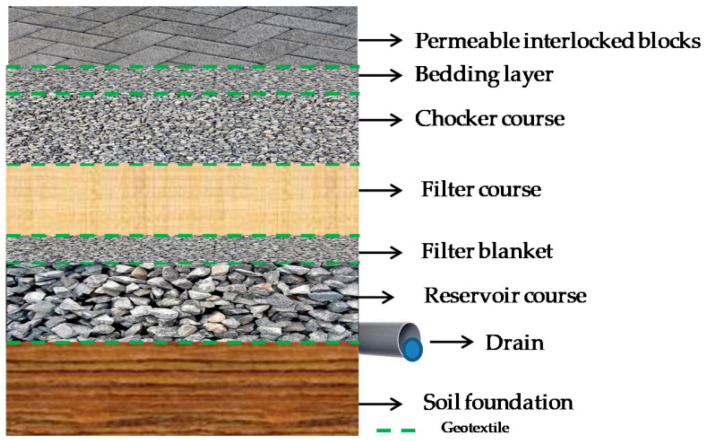
Cross-section of a permeable interlocking concrete pavement [11].

For example, in the PICP system, rainwater infiltrates through the joints between the pavers, into the bedding layer, and then into the sand filter [9,10]. Figure 1 shows a typical cross-section of the PICP system [11]. As rainwater passes through the sand filter, pollutants such as sediment and hydrocarbons are removed. The filtered water can then infiltrate into the natural groundwater, where it can recharge the groundwater reservoir. The sand filter can be an effective way to improve the quality of rainwater runoff.

A study was conducted by Niu et al. [12] to evaluate the rainwater infiltration and runoff pollution reduction performance of permeable pavements, including permeable interlocking blocks, bedding layers (thickness = 2, 3.5, and 5 cm), and open-graded layers (thicknesses = 15, 20, 25, and 30 cm). The results indicated that permeable pavement layer thickness was an important factor influencing the infiltration and pollution removal rates of the permeable pavement. In addition, Ghisi [13] evaluated the filtration performance of the PAP and the quality of rainwater filtered by filter layers. The results showed that the PAP is able to filter pollutants from rainwater.

For permeability, Arafa et al. [14] conducted an investigation of the permeability of permeable geopolymer concrete (PGC) to determine the rainwater runoff reduction of PGC. The results showed that the PGC was an effective means for reducing runoff. In addition, Praticò and Moro [15] carried out a study with a theoretical and experimental analysis of the percolation phenomena of water in porous asphalt concrete (PAC). It was concluded that effective porosity was a very important factor when analyzing permeability.

Permeable pavements are susceptible to clogging, which occurs when sediment fills the voids in the aggregate layers, reducing the system’s effectiveness in managing rainwater runoff. A study by Lucke and Beecham [8] investigated the clogging behavior of permeable pavement, which is designed to reduce rainwater runoff and improve water quality by allowing rainwater to infiltrate into the ground. Their experimental tests showed that clogging occurred at the interface between blocks and bedding aggregates, reducing infiltration capacity over time. Ghisi et al. [11] evaluated the ability of PICP to filter rainwater for non-potable uses in buildings and found that a sand filter layer effectively reduced pollutant levels. In addition, Marcaida et al. [16] conducted laboratory experiments to investigate factors contributing to particle-related clogging in pervious concrete and found that mixes with high proportions of fine particles clogged more quickly than those with more coarse particles. 

Permeable pavements are designed to reduce rainwater runoff and improve water quality, but the accumulation of TSS in pavement voids can cause clogging and reduce infiltration rates. TSS refers to the concentration of solid particles that are suspended in water and can be measured as a water quality parameter [17,18]. These TSS and pollutants from rainwater runoff are directly filtered by permeable pavements during rainfall events. Over time, TSS gradually accumulates in the pavement layers, and the infiltration capacity is reduced [19,20].

The filter layer in the permeable pavement is a crucial factor to consider for preventing permeability degradation and enhancing TSS removal efficiency. Thus, the aim of this study is to investigate three factors that impact the permeability degradation and TRE of the sand filter in the permeable pavement: TSS particle size, TSS concentration, and hydraulic gradient. Eight tests were conducted using varying values of these factors to accomplish the goal.

## 2. Materials and Methods

The permeability degradation and TSS removal efficiency were investigated based on two sets of laboratory experiments. In the first set of experiments, the permeability of the sand filter before clogging was investigated. In the second set of experiments, the water mixed with TSS was applied to the sand filter, and both permeability degradation and TSS removal efficiency were evaluated in parallel.

### 2.1. Sand Filter and TSS Materials

**Figure 2:**
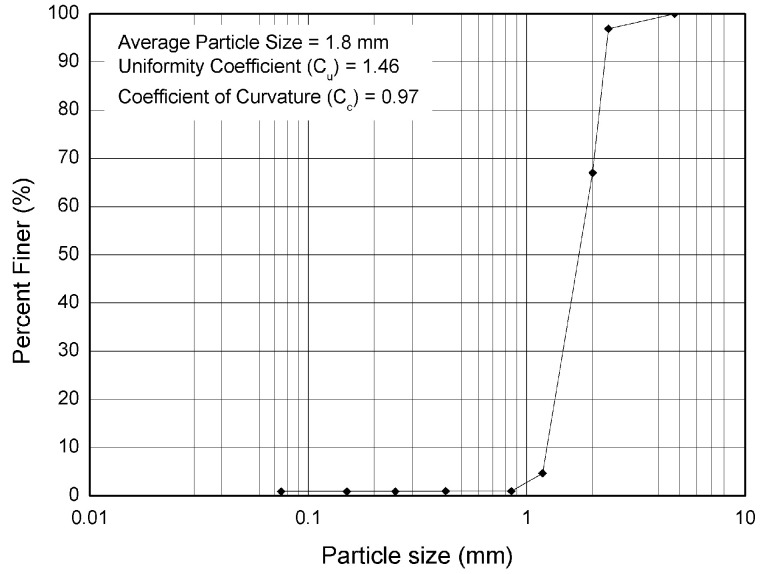
Sand filter particle size distribution curve.

In this study, silica sand was selected as a filter material, which is a typical application for permeable pavement systems. The particle size distribution curve of the silica sand is shown in Figure 2. The average particle size of the sand is 1.8 mm. The gradation is uniform with the coefficients of uniformity (1.46) and curvature (0.97).

**Figure 3:**
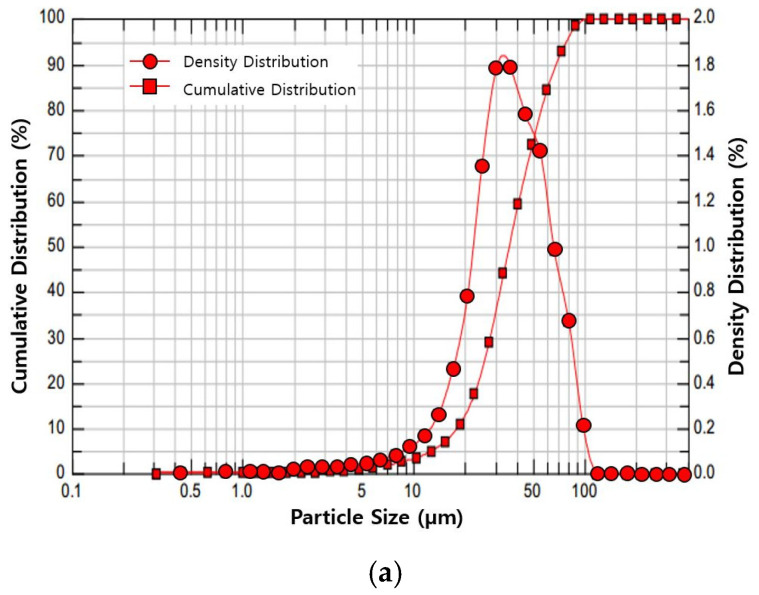

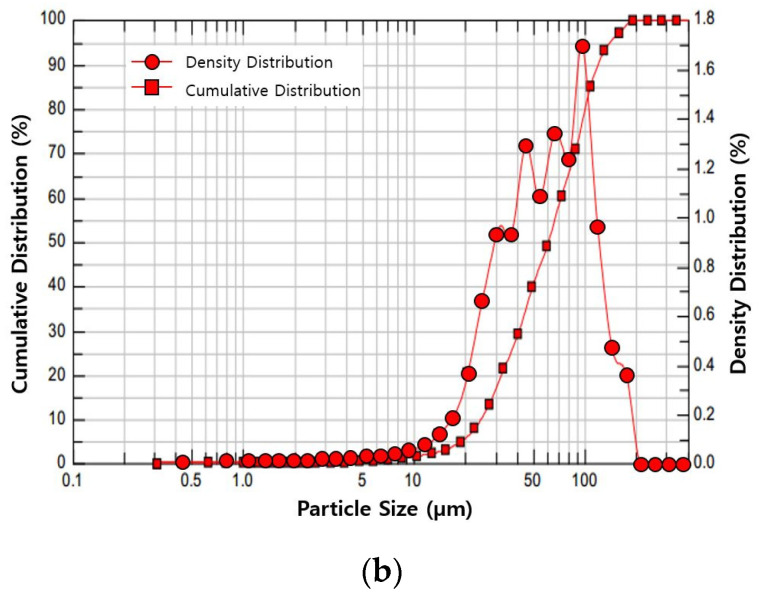
Distribution of TSS particle sizes. (**a**) Particle size distribution of 0.035 mm; (**b**) particle size distribution of 0.060 mm.

The 0.035-mm and 0.060-mm granular particles were used as TSS. In this study, permeability degradation and TSS removal efficiency are evaluated in parallel for a single sand filter sample, and therefore TSS and clogging particles represent the same material. Figure 3 shows the gradation curves of the TSS materials analyzed using an off-line image analyzer, QICPIC, and software WINDOX 5.9.0.0 [21].

### 2.2. Evaluation of Permeability 

**Figure 4:**
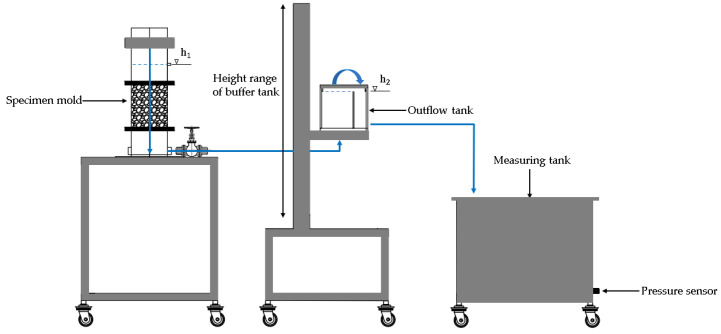
Schematic of test equipment [22].

**Figure 5:**
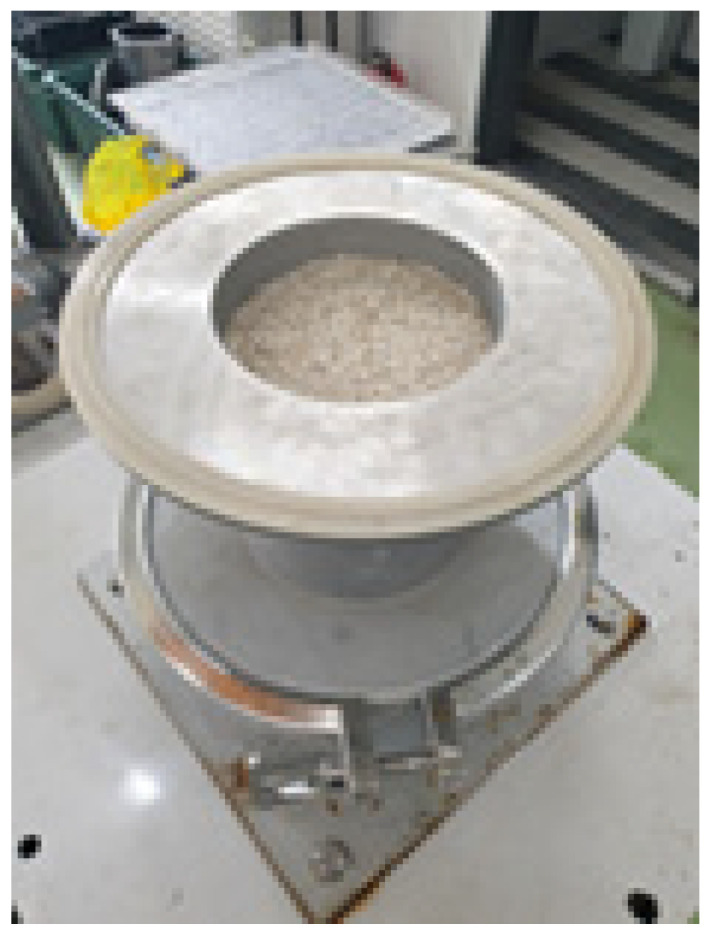
A specimen in the sample mold.

The test equipment developed by Ahn et al. [22] was employed to evaluate the permeability. Figure 4 shows an overview of the equipment. This equipment can be operated under constant head differences during the test. The head difference can be varied by raising or lowering the level of the outlet vessel. It should be noted that for some porous materials, particularly those with large pores, the flow rate and hydraulic gradient do not necessarily have a linear relationship. From the volume of water recorded over time in the measuring tank, the flow rate of the water passing through the system can be calculated. An example of the silica sand in the sample mold of the apparatus is shown in Figure 5.

According to Darcy’s law, the relationship between the discharge velocity (v) and the hydraulic gradient (i) can be presented as:

v = ki,(1)

where k is the permeability and the slope of the velocity-hydraulic gradient plot. For some permeable pavement materials, Darcy’s law does not apply, but a non-linear relationship between discharge velocity and the hydraulic gradient is more reliable [23,24]:

v = ki^n^, (2)

where n is an exponent to account for non-linearity. For laminar flow, the variable n is 1, while for turbulent flow, the variable n is 0.5 [25,26]. Four different hydraulic gradients (0.5, 1.0, 1.5, and 2.0) were used in this study.

### 2.3. Evaluation of Permeability Degradation and TSS Removal Efficiency

**Figure 6:**
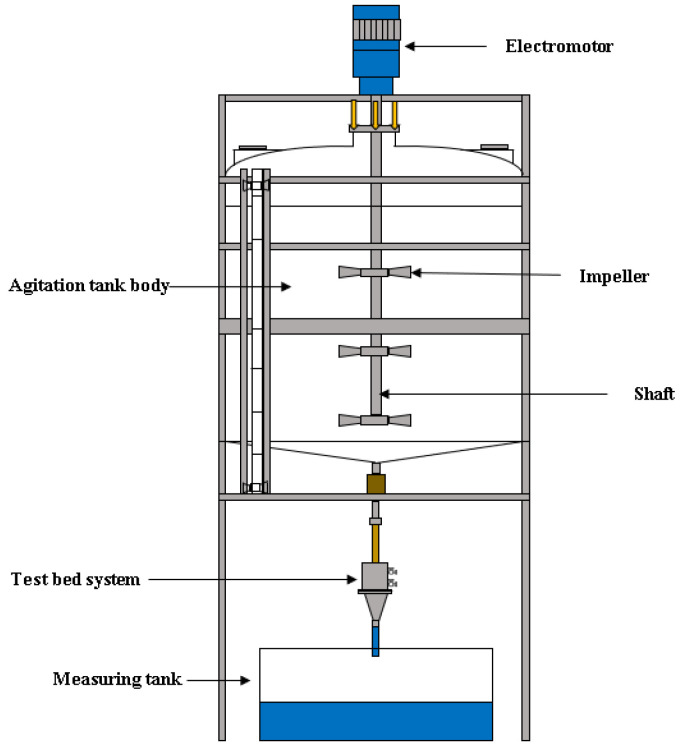
Permeability degradation and TSS removal equipment [28].

In order to evaluate the degradation of the permeability of the sand filter and the removal of TSS in parallel, the equipment shown in Figure 6 is selected and used. The water contaminated with TSS (0.035 mm or 0.060 mm) is prepared in an agitation tank. Based on Memon et al., two different concentrations of TSS, 100 mg/L and 300 mg/L, were targeted in this study [27]. The mixed water is discharged through an outlet pipe into the infiltration cell, where the sand specimen is placed.

**Figure 7:**
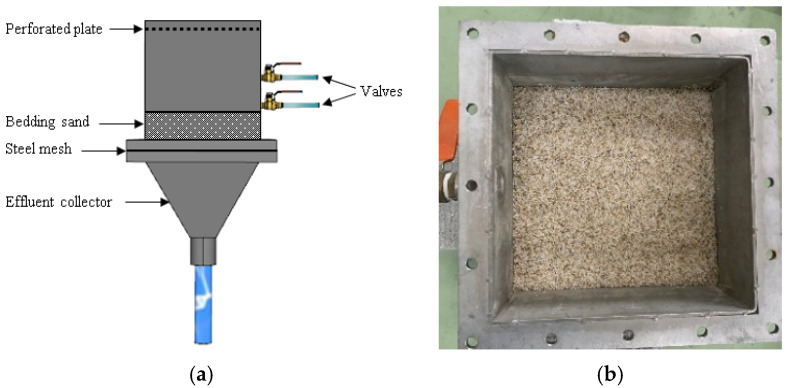
(**a**) Schematics of the test bed system; (**b**) sand filter in the infiltration cell.

The infiltration cell, located below the agitator tank, is a three-layer steel column with a cross-section of 200 × 200 mm and a tapered end, as shown in Figure 7a. The first layer receives the mixed water from the tank, and the water level is maintained by outlet values. The second layer contains a specimen, the sand filter (Figure 7b). The sand was washed and dried prior to installation in order to minimize the fines discharged from the sand specimen itself. The last layer, the tapered layer, collects the water and conveys it to the measurement tank, where the flow rate is estimated.

The experiments were conducted with two different sizes of TSS particles (0.035 mm and 0.060 mm), two TSS concentrations (100 mg/L and 300 mg/L), and two hydraulic gradients (1.1 and 2.0), which makes a total of eight test cases. Table 1 summarizes the test cases and the corresponding TSS particles, TSS concentrations, and hydraulic gradients selected.

Inlet and outlet water samples were taken every 10 min and analyzed according to “ Standard Methods for the Examination of Water and Wastewater” [29]. The TRE can be calculated as:

(3) $$\mathrm{TRE}\;\left(\%\right)=\frac{{\mathrm{TSS}}_{\mathrm{in}}-{\;\mathrm{TSS}}_{\mathrm{out}}}{{\mathrm{TSS}}_{\mathrm{in}}}\times 100,$$

where ${\mathrm{TSS}}_{\mathrm{in}}$ is the inflow TSS and ${\mathrm{TSS}}_{\mathrm{out}}$ is the outflow TSS.

## 3. Result and Discussion

### 3.1. Permeability

**Figure 8:**
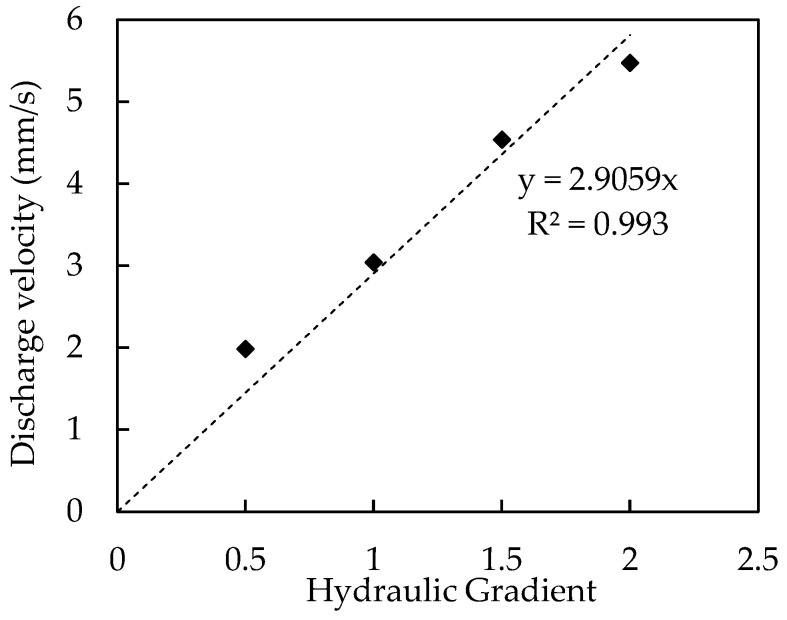
Relationship between discharge velocity and hydraulic gradient of silica sand.

The measured discharge velocity is plotted against the corresponding hydraulic gradient in Figure 8. It is well known that Darcy’s Law applies to typical soil materials, which includes the silica sand tested here. A linear regression analysis was carried out to evaluate the permeability of the sand filter material. The slope of this relationship is equivalent to the permeability coefficient of the sand filter. Based on the analysis, the permeability was estimated to be 2.91 mm/s, with a coefficient of determination of R^2^ = 0.993.

### 3.2. Permeability Degradation

**Figure 9:**
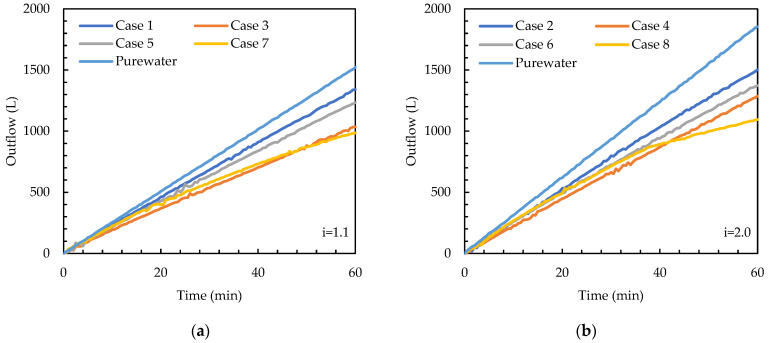
Volume of outflow over time. (**a**) i = 1.1; (**b**) i = 2.0.

This study investigated the effects of TSS particle size, TSS concentration, and hydraulic gradient on permeability degradation. Figure 9 displays the volume of outflow over time, which shows degradation of permeability, for the tests conducted. For 60 min, which is the test duration, the outflow gradually increases over time. The outflow volume was measured with a pressure sensor, and the sensitivity of the sensor was reflected in the curve of flow volume (fluctuation). The attenuation of outflow and, therefore, the degradation of permeability over time may be influenced by TSS particle size, TSS concentration, and hydraulic gradient. When the flow rates of pure water are investigated for the target hydraulic gradients of 1.1 and 2.0, they are not proportional to the target gradients. It is noted that there is suction developed at the interface of the saturated soil and the air placed at the bottom of the specimen, which develops larger hydraulic gradients than the target values. 

According to Figure 9, for the test cases analyzed, the application of the 0.060 mm TSS particles results in more permeability degradation than the 0.035 mm particles. For example, for i = 1.1, Cases 3 and 7 (0.060 mm particles) show a lower outflow than Cases 1 and 5 (0.035 mm particles), regardless of the concentration used (Figure 9a). The same trend is also observed for i = 2.0 (Figure 9b). This is because smaller particles (0.035 mm) can pass through the voids of the filter material better than larger particles (0.060 mm).

From Figure 9, it appears the TSS concentration and hydraulic gradient also have effects on the degradation of permeability. For i = 1.1, Cases 1 and 3 (100 mg/L concentration) result in higher outflow than Cases 5 and 7 (300 mg/L concentration) (Figure 9a). The same trend is also observed for i = 2.0 (Figure 9b). In addition, the outflow attenuation of four test cases from pure water with a larger hydraulic gradient (Figure 9b) is greater than that of those with a smaller hydraulic gradient (Figure 9a). Overall, the effect of TSS concentration and hydraulic gradient on permeability degradation is not as significant as the effect of TSS particle size.

### 3.3. TSS Removal Efficiency

**Figure 10:**
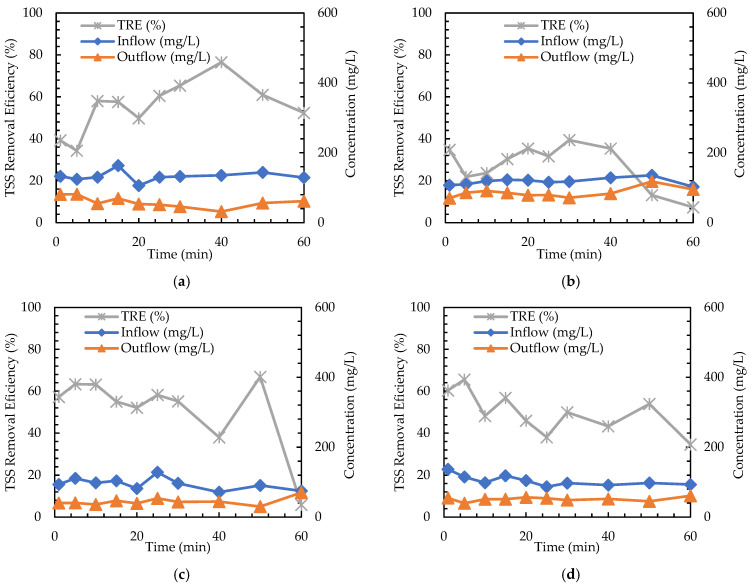

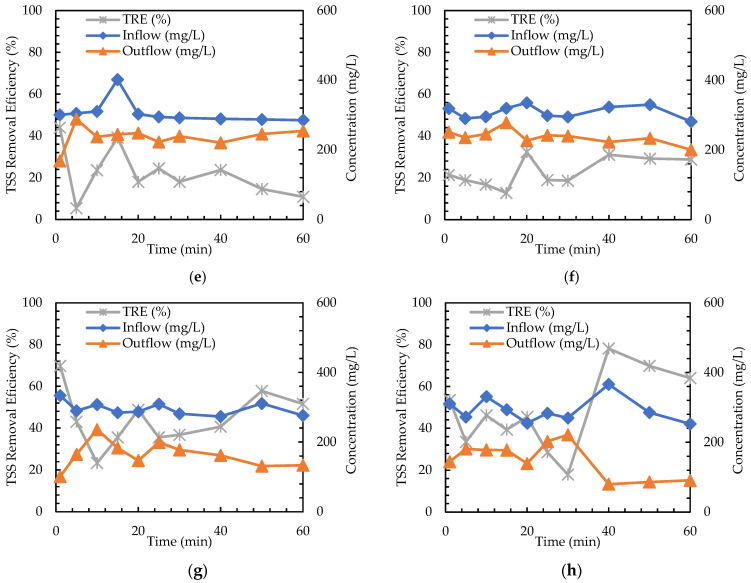
TRE results. (**a**) Case 1: 0.035 mm, i = 1.1; 100 mg/L; (**b**) Case 2: 0.035 mm, i = 2.0; 100 mg/L; (**c**) Case 3: 0.060 mm, i = 1.1; 100 mg/L; (**d**) Case 4: 0.060 mm, i = 2.0; 100 mg/L; (**e**) Case 5: 0.035 mm, i = 1.1; 300 mg/L; (**f**) Case 6: 0.035 mm, i = 2.0; 300 mg/L; (**g**) Case 7: 0.060 mm, i = 1.1; 300 mg/L; (**h**) Case 8: 0.060 mm, i = 2.0; 300 mg/L.

Two independent sets of experiments were carried out to evaluate the TSS removal efficiency of the filter specimen. A sample set of the test results is shown in Figure 10, which shows the inflow concentration, outflow concentration, and TRE estimated from measurements with intervals.

**Figure 11:**
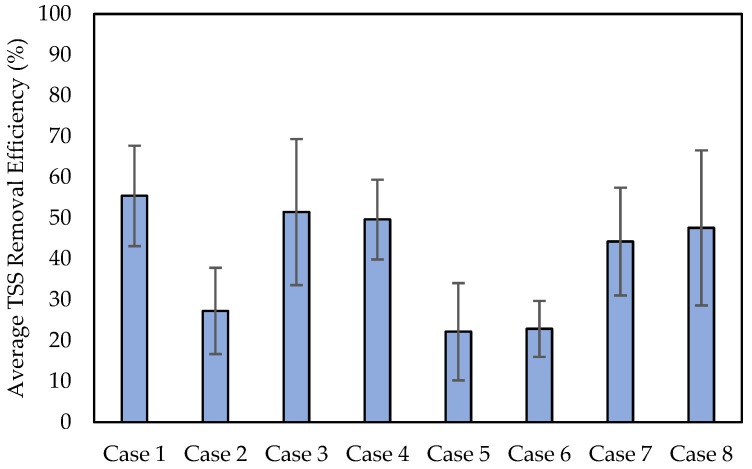
The average TSS removal efficiency.

A total of 16 measurements of TSS removal efficiency were obtained from two sets of tests. The average TSS removal efficiency of the measurements is summarized in Figure 11. The graph shows that the TSS particle size is the main factor influencing TRE. Specifically, the application of 0.060 mm TSS particles (Cases 3, 4, 7, and 8) results in high TRE. This is because the smaller TSS particles (0.035 mm) can pass through the voids of the filter better than the larger TSS particles (0.060 mm).

The effect of TSS concentrations and hydraulic gradients on TRE was also investigated. For Cases 1 and 5, the lower TSS concentration (100 mg/L) in Case 1 results in a higher TRE, while the higher TSS concentration (300 mg/L) in Case 5 leads to a lower TRE. Additionally, TRE is also investigated for two target hydraulic gradient values of 1.1 and 2.0. For Cases 1 and 2, the smaller hydraulic gradient results in a higher TRE. It appears that a larger discharge velocity associated with a larger hydraulic gradient makes the TSS particles less clogged when passing through the filter specimen. Overall, the effect of TSS concentration and hydraulic gradient on TRE is not as significant as the effect of TSS particle size in the cases of the experiments conducted. Among the cases with 0.035 mm particles (Cases 1, 2, 5, and 6), only Case 1 with a TSS concentration of 100 mg/L and a target hydraulic gradient of 1.1 results in a higher TRE than the others.

## 4. Conclusions

This study aims to investigate the permeability degradation and TSS removal efficiency of sand filters in permeable pavement. By conducting a series of tests, the effect of three factors—TSS particle size, TSS concentration, and hydraulic gradient—on permeability degradation and TSS removal efficiency was investigated. From the results of the experiments reported in this study, the following conclusions can be drawn:

- TSS particle size has a significant effect on permeability degradation. It appears that larger TSS particles (0.060 mm) can lead to greater permeability degradation than smaller TSS particles (0.035 mm). This is because smaller particles are more likely to pass through the filter voids better than larger particles, whereas larger ones are not.
- The degradation of permeability appears to be influenced by both the TSS concentration and the hydraulic gradient. Specifically, a higher TSS concentration (300 mg/L) and a smaller hydraulic gradient (1.1) result in higher permeability degradation. However, their influence is not as significant as that of TSS particle size.
- TSS particle size also influences TSS removal efficiency. Specifically, it appears that the larger TSS particles (0.060 mm) can lead to higher TRE compared to the smaller TSS particles (0.035 mm), which is consistent with the results of permeability degradation.
- It is observed that TRE generally tends to be higher at a lower TSS concentration (100 mg/L) and a smaller hydraulic gradient (1.1). TSS concentration and hydraulic gradient may influence TRE, although their influence may be less significant than that of TSS particle size.

## Figures and Tables

**Table 1:** Test cases.

No.	TSS Particle Size, mm	TSS Concentration, mg/L	Hydraulic Gradient
Case 1	0.035	100	1.1
Case 2	0.035	100	2.0
Case 3	0.060	100	1.1
Case 4	0.060	100	2.0
Case 5	0.035	300	1.1
Case 6	0.035	300	2.0
Case 7	0.060	300	1.1
Case 8	0.060	300	2.0

## Data Availability

Not applicable.

## References

[1] Booth D.B., Leavitt J. (1999). Field Evaluation of Permeable Pavement Systems for Improved Stormwater Management. J. Am. Plan. Assoc..

[2] Davis A.P., Shokouhian M., Ni S. (2001). Loading estimates of lead, copper, cadmium, and zinc in urban runoff from specific sources. Chemosphere.

[3] Konrad C.P. (2003). Effects of Urban Development on Floods.

[4] Coffman L.S. (2002). Low-Impact Development: An Alternative Stormwater Management Technology.

[5] Dietz M.E. (2007). Low impact development practices: A review of current research and recommendations for future directions. Water Air. Soil Pollut..

[6] Field R., Masters H., Singer M. (1982). Status of porous pavement research. Water Res..

[7] Marchioni M., Becciu G. (2014). Permeable pavement used on sustainable drainage systems (SUDs): A synthetic review of recent literature. WIT Transactions on the Built Environment.

[8] Lucke T., Beecham S. (2011). Field investigation of clogging in a permeable pavement system. Build. Res. Inf..

[9] Nichols P.W.B., Lucke T., Dierkes C. (2014). Comparing Two Methods of Determining Infiltration Rates of Permeable Interlocking Concrete Pavers. Water.

[10] Lucke T., White R., Nichols P., Borgwardt S. (2015). A Simple Field Test to Evaluate the Maintenance Requirements of Permeable Interlocking Concrete Pavements. Water.

[11] Ghisi E., Belotto T., Thives L. (2020). The Use of Permeable Interlocking Concrete Pavement to Filter Stormwater for Non-Potable Uses in Buildings. Water.

[12] Niu Z.-G., Lv Z.-W., Zhang Y., Cui Z.-Z. (2016). Stormwater infiltration and surface runoff pollution reduction performance of permeable pavement layers. Environ. Sci. Pollut. Res..

[13] Ghisi E. (2018). Filtering Capability of Porous Asphalt Pavements. Water.

[14] Arafa S., Milad A., Yusoff N.I.M., Al-Ansari N., Yaseen Z.M. (2021). Investigation into the permeability and strength of pervious geopolymer concrete containing coated biomass aggregate material. J. Mater. Res. Technol..

[15] Praticò F.G., Moro A. (2007). Permeability and Volumetrics of Porous Asphalt Concrete. Road Mater. Pavement Des..

[16] Marcaida A., Nguyen T., Ahn J. (2018). Investigation of Particle-Related Clogging of Sustainable Concrete Pavements. Sustainability.

[17] Sartor J.D., Boyd G.B., Agardy F.J. (1974). Water pollution aspects of street surface contaminants. J. Water Pollut. Control Fed..

[18] Ismail A.F., Khulbe K.C., Matsuura T. (2019). RO Membrane Fouling. Reverse Osmosis.

[19] Pezzaniti D., Beecham S., Kandasamy J. (2009). Influence of clogging on the effective life of permeable pavements. Proc. Inst. Civ. Eng. Water Manag..

[20] Field R., Masters H., Singer M. (1982). An overview of porous pavement research. J. Am. Water Resour. Assoc..

[21] List J., Köhler U., Witt W. (2011). Dynamic Image Analysis extended to Fine and Coarse Particles. Part. Syst. Anal..

[22] Ahn J., Maricris J., Shin H.-S., Jung J. (2017). Test Equipment and Procedure to Evaluate Permeability Characteristics of Permeable Pavements. Korean Soc. Hazard Mitig..

[23] Coleri E., Kayhanian M., Harvey J.T., Yang K., Boone J.M. (2013). Clogging evaluation of open graded friction course pavements tested under rainfall and heavy vehicle simulators. J. Environ. Manag..

[24] Liu R., Liu H., Sha F., Yang H., Zhang Q., Shi S., Zheng Z. (2018). Investigation of the Porosity Distribution, Permeability, and Mechanical Performance of Pervious Concretes. Processes.

[25] Fwa T.F., Tan S.A., Guwe Y.K. (1999). Laboratory Evaluation of Clogging Potential of Porous Asphalt Mixtures. Transp. Res. Rec. J. Transp. Res. Board.

[26] Huang B., Mohammad L.N., Raghavendra A., Abadie C. (1999). Fundamentals of permeability in asphalt mixtures. Proc. Assoc. Asph. Paving Technol..

[27] Memon S., Go S., Lee C.-H. (2013). Evaluation of First Flush Phenomenon from Bridge and Parking Lot Sites in the Gyeongan Watershed in Korea. Water Environ. Res..

[28] Kim J., Choi Y., Kim T., Lee S.H., Kwon S. (2019). Study on the Permeability and TSS Removal Efficiency of Permeable Pavement Using Constant Head Particle Loading Test. J. Coast. Res..

[29] Bridgewater L., American Public Health Association, American Water Works Association (1998). Water Environment Federation. Standard Methods for the Examination of Water and Wastewater.

